# Generating a 30 m Hourly Land Surface Temperatures Based on Spatial Fusion Model and Machine Learning Algorithm

**DOI:** 10.3390/s24237424

**Published:** 2024-11-21

**Authors:** Qin Su, Yuan Yao, Cheng Chen, Bo Chen

**Affiliations:** 1 School of Architecture and Civil Engineering, Chengdu University, Chengdu 610106, China; suqinchongqing@aliyun.com (Q.S.); hnchencheng@aliyun.com (C.C.); chenbo@cdu.edu.cn (B.C.); 2 Key Laboratory of Pattern Recognition and Intelligent Information Processing of Sichuan Province, Chengdu University, Chengdu 610106, China; 3 College of Earth Science, Chengdu University of Technology, Chengdu 610059, China

**Keywords:** land surface temperature, Chinese geostationary weather satellite, multi-scale polar-orbiting satellite, spatiotemporal fusion model

## Abstract

Land surface temperature (LST) is a critical parameter for understanding climate change and maintaining hydrological balance across local and global scales. However, existing satellite LST products face trade-offs between spatial and temporal resolutions, making it challenging to provide all-weather LST with high spatiotemporal resolution. In this study, focusing on Chengdu city, a framework combining a spatiotemporal fusion model and machine learning algorithm was proposed and applied to retrieve hourly high spatial resolution LST data from Chinese geostationary weather satellite data and multi-scale polar-orbiting satellite observations. The predicted 30 m hourly LST values were evaluated against in situ LST measurements and Sentinel-3 SLSTR data on 11 August 2019 and 21 April 2022, respectively. The results demonstrate that validation based on the in situ LST, the root mean squared error (RMSE) of the predicted LST using the proposed framework are around 0.89 °C to 1.23 °C. The predicted LST is highly consistent with the Sentinel-3 SLSTR data, and the RMSE varies from 0.95 °C to 1.25 °C. In addition, the proposed framework was applied to Xi’an City, and the final validation results indicate that the method is accurate to within about 1.33 °C. The generated 30 m hourly LST can provide important data with fine spatial resolution for urban thermal environment monitoring.

## 1. Introduction

Land surface temperature (LST) is a key variable in the surface radiation budget, as it, along with surface emissivity, governs the upward thermal radiation, which reflects energy and water exchange between the land surface and the atmosphere [1,2]. Accordingly, LST plays a vital role in numerous research fields, for example, the surface urban heat island (SUHI) [3,4], hydrology [5,6], climate change [7,8], urban planning [9], forest fire detection [10], surface energy balance [11], and evapotranspiration estimations [12,13,14]. Gaining new insights into LST can enhance our understanding of the surface radiation budget and land surface-atmosphere exchange processes from regional to global scales. Therefore, the Global Climate Observing System has designated LST as one of the 10 essential climate variables within the land biosphere [15]. Compared to ground measurements, satellite-derived LST offers the unique advantage of monitoring LST from a regional to a global scale. High spatiotemporal resolution LST data enables researchers to identify UHI hotspots at a finer scale and develop targeted mitigation strategies, such as increasing green space coverage and improving urban building materials, to effectively reduce the impact of the UHI effect [16]. Additionally, it enhances the understanding of hydrological processes by providing valuable insights into energy fluxes, evapotranspiration, soil moisture, and water distribution, thereby supporting hydrological studies and resource management, particularly in arid and semi-arid regions [17]. Furthermore, it helps analyze the frequency, spatiotemporal distribution, and intensity of extreme weather events (such as heatwaves and cold waves), offering critical data support for disaster warning and response [18].

The potential application of satellite-derived LST products is limited by the trade-offs in satellite capabilities: polar-orbiting satellites offer high spatial resolution but relatively low temporal resolution, while geostationary satellites provide high temporal resolution but have lower spatial resolution. For example, polar-orbiting satellites like the Moderate-Resolution Imaging Spectroradiometer (MODIS) can generate daily LST data with a spatial resolution of 1 km, while Landsat 8 and Landsat 9 offer LST data with a spatial resolution of 100 m but have a temporal resolution of 16 days. This temporal resolution is insufficient for effectively monitoring and predicting urban thermal environment dynamics [19,20,21]. In addition, Geostationary (GEO) satellite sensors such as Geostationary Operational Environmental Satellite (GOES) provide official LST data with a spatial resolution of 4 km coverage of full earth disk approximately half-hourly but experience a midnight effect that can result in erroneous instrument responsivity around satellite midnight, which result in a large mean brightness temperature (Tb) bias [19]. Although the Midnight Blackbody Calibration Correction (MBCC) method has been reported to improve midnight calibration in short-wave channels [22], recent inter-calibration analyses between the GOES Imager and new low-Earth-orbit instruments show a significant Tb difference remains in some GOES Imager long-wave channels around satellite midnight [23,24]. The FY-4A satellite, launched by China in 2016, is a second-generation geostationary meteorological satellite with advanced instruments for Earth observation. It provides high-precision, multi-spectral, and high-frequency data for weather, disaster prevention, land-atmosphere research, and climate change [25]. FY-4A provides official LST data with a spatial resolution of 4 km in NETCDF format, with coverage every 15 min or better. Dong et al. [26] inverted and validated the FY-4A official LST product using in situ measured LST data and MODIS LST products. Their results showed that the general root mean square error (RMSE) of the FY-4A LST product, after preprocessing, is better than 3 K. Li et al. [27] utilized FY-4A LST data along with auxiliary variables to reconstruct missing LST data. The experimental results demonstrate that the RMSE of the reconstructed LST, under both theoretical clear-sky and cloudy conditions, remains within 2.9 K. The key advantage of the FY-4A over polar-orbiting satellites is its ability to provide higher-frequency LST observations, including diurnal variation, for regions around China. This data is crucial for assessing the impact of hourly-scale LST on climate models [28]. Therefore, developing a method to generate high spatiotemporal resolution LST is crucial, as it would greatly benefit urban thermal environment monitoring.

Spatiotemporal fusion methods provide a feasible solution for generating high spatiotemporal resolution satellite images, overcoming the limitations of current satellites [29,30,31]. Previous work has classified typical spatiotemporal data fusion methods into five groups: weight function-based methods (e.g., STARFM [32], Bilateral Filter [33], STITFM [34], Fit-FC [35]), unmixing-based methods (e.g., MMT [36], STDFA [37], pMSRAFM [38]), Bayesian-based methods (e.g., BMF [39], Unified fusion [40], Bayesian data fusion approach [41]), learning-based methods (e.g., DPSTFN [42], SPSTFM [43], HCNNet [44], STFDCNN [45]), and hybrid methods (e.g., STRUM [46], STIMFM [47], FSDAF [48]). Weight function-based methods are widely used, especially for LST, due to their simplicity, robustness, and high efficiency [49]. However, they struggle to capture instantaneous and shape changes, and their performance is sensitive to model parameters, particularly the moving window size, often resulting in RMSE exceeding 1 K [50]. Unmixing-based methods are seldom used in LST spatiotemporal fusion studies and will not be discussed here. Bayesian-based methods offer distinct advantages for LST prediction, especially when fusing data from different observation methods [33], but their construction is complex and abstract. Learning-based methods use machine learning techniques to generate high spatiotemporal resolution LST images, but their performance is dependent on training intensity. The limited spectral characteristics of LST make accumulating sufficient training data challenging, often leading to poorer fusion performance compared to traditional weight function-based methods [51]. Hybrid methods combine multiple models to leverage their strengths, optimizing algorithm structure and improving accuracy, making them the most widely used approach in spatiotemporal LST fusion studies [52].

While many methods have been proposed to generate high spatiotemporal resolution LST, most focus on fusing data from two different polar-orbiting satellites [53,54]. Few studies have successfully combined geostationary and multi-scale polar-orbiting satellites to produce hourly LST data with high spatial resolution, which is essential for applications like urban thermal environment monitoring at a regional scale. This primarily arises from two factors: first, time and spatial consistency—while geostationary satellites deliver continuous LST data, polar-orbiting satellites provide intermittent LST data with limited coverage [55]. To combine these LST datasets, it’s crucial to address both temporal and spatial consistency to ensure that LST variations align within the same time frame. Second, differences in LST data quality and accuracy—the LST retrieval methods of the two satellite types differ, which can lead to discrepancies in accuracy and error characteristics [56].

This study focuses on Chengdu City in southwest China, an area where urban expansion has significantly contributed to the deterioration of the urban thermal environment [57]. This study develops a novel fusion framework that combines a comprehensive, flexible spatiotemporal data fusion (CFSDAF) method with a machine learning algorithm to predict reliable high spatial resolution hourly LST data. The framework integrates data from geostationary satellites (FY-4A LST) and multi-scale polar-orbiting satellites (MODIS LST and Landsat LST). In situ, LST measurements and Sentinel-3 SLSTR data were used to evaluate the predicted LST under both cloudy and clear conditions. This framework involves two main steps: (1) predicting hourly LST data at 1 km spatial resolution using MOD11A1 and FY-4A LST data, and (2) combining the predicted results and downscaled LST data to generate hourly LST data at 30 m spatial resolution.

## 2. Data and Methods

### 2.1. Study Area

**Figure 1:**
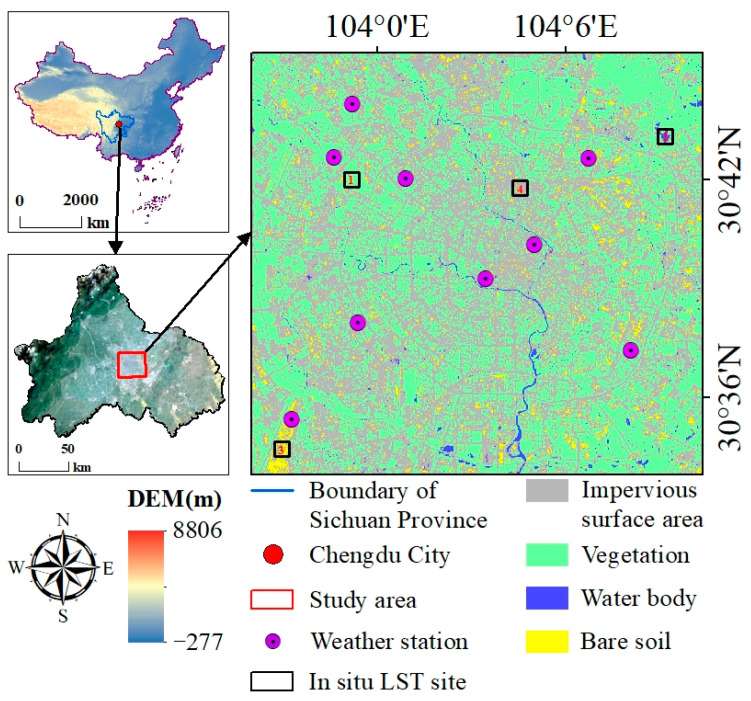
Location and land-cover maps of the study area.

The study area is located in the center of Chengdu, Sichuan Province, in southwestern China, with coordinates ranging from 103°55′45″ E to 104°10′47″ E and 30°33′52″ N to 30°45′32″ N. Chengdu, characterized by a typical subtropical monsoon humid climate, experiences frequent rainfall throughout the year. From a land-cover perspective, impervious surface areas (ISA) dominate, primarily due to the urban expansion of Chengdu. The four mainly land-cover types in the study area are ISA, vegetation, bare soil, and water bodies. The land cover types in the study area are diverse, which is significant for testing whether the predicted LST data, based on the proposed framework, is applicable to heterogeneous areas. In this study, a support vector machine (SVM) classifier [58] was used for land-cover classification (Figure 1). The radial basis function (RBF) kernel of the SVM classifier was used to implement the SVM algorithm by ENVI software version 6.0 for land cover mapping.

**Figure 2:**
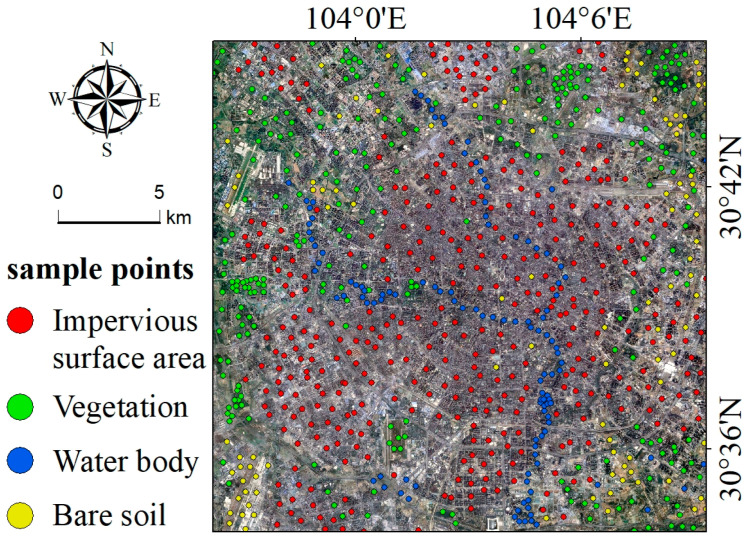
Sample points from the Google Earth image used to validate the accuracy of land cover classification.

For an accurate assessment of the classification results, two high-resolution satellite images with a 1 m spatial resolution from Google Earth, captured in 2019 and 2022, were utilized. A total of 1000 sample points representing all land cover types (Figure 2) were collected from the Google Earth imagery to evaluate the accuracy of the classification results. The reliability of land cover classification performed using the SVM method was validated using the producer’s accuracy, user’s accuracy, overall accuracy, and Kappa statistics (Table 1).

Table 1 demonstrates that the overall accuracies for both land cover types exceeded 90%, with Kappa statistics well above 0.85. Similarly, the producer’s accuracy for each land cover type ranged from 0.90 to 0.95, while the user’s accuracy ranged from 0.87 to 0.93, indicating strong agreement between the classification map and the ground reference data. Furthermore, more than 90% of the study area was composed of vegetation and impervious surface area (ISA) in both 2019 and 2022. The proportion of ISA increased significantly, from 46.00% in 2019 to 57.70% in 2022, while the percentage of vegetation decreased from 49.30% to 39.60% during the same period. These findings suggest that Chengdu continues to experience rapid urban expansion.

### 2.2. Data Description and Preprocessing

The primary data used in this study included FY-4A LST, MOD11A1, Landsat LST, and in situ LSTs. Table 2 shows the details of the LST dataset used. Landsat LST data was obtained from the TIR band at 100 m spatial resolution. Notably, in the USGS Landsat Level 2 product, the TIR band has been resampled to 30 m to align with the optical bands; however, its actual spatial resolution remains 100 m [59].

(1) FY-4A LST data. The FY-4A satellite is a new-generation Chinese geostationary meteorological satellite, which successfully launched in 2016. The FY-4A LST data, with a temporal resolution of 15 min and a spatial resolution of 4 km, is derived using the split-window algorithm and can be downloaded from the Fengyun Satellite Remote Sensing Data Network (http://satellite.nsmc.org.cn/ accessed on 1 August 2024). In this study, full-disk 4 km GEO data was used to create a latitude and longitude lookup table, enabling the reprojection of disk data into the nominal geostationary orbit projection based on the WGS84 reference ellipsoid. This was followed by batch geometric correction of the FY-4A LST data.

(2) MODIS LST data. MOD11A1 (version 6) is a 1 km daily MODIS LST product retrieved using a generalized split-window algorithm with an accuracy better than 1 °C [60]. MOD11A1, with an overpass time of around 11:00 local time, is available from the NASA Earth data Search platform (https://search.earthdata.nasa.gov/search accessed on 2 August 2024). In this study, the MODIS Reprojection Tools was used to re-project the data to match the coordinate system of the Universal Transverse Mercator system (UTM/WGS84).

(3) Landsat LST data. Landsat TIR channels have a spatial resolution of 100 m, but the revisiting period of 16 days. Cloud-free Landsat data was downloaded from the U.S. Geological Survey (http://earthexplorer.usgs.gov/ accessed on 2 August 2024). In this study, the mono-window algorithm [61] was used to retrieve LST data after atmospheric correction. Surface emissivity in Landsat TIR band 10 is a key input parameter for retrieving LST. Emissivity data for water, bare soil, ISA, and vegetation were obtained from the ASTER Global Emissivity Dataset (100-m, Version 003) and resampled to a 30 m spatial resolution using cubic interpolation. In this study, average emissivity values of 0.99683 for water, 0.96767 for soil, 0.96488 for ISA, and 0.98672 for vegetation were applied.

(4) In situ measured LST data. The ground-based in situ measured LST data consists of two parts. The first part was obtained from nine weather stations by the Chengdu Meteorological Office, where all LST data were automatically observed using SI-111 infrared radiometers with an accuracy of ±0.2 °C. The in situ LST ($T_{i}$) is obtained as follows [62]:

(1) $$T_{i}=B^{-1}[\frac{T_{s}-\left(1-\varepsilon \right)L^{↓}}{\varepsilon }]$$

where $T_{s}$ is the radiance measured by the SI-111 radiometer, *B* is the Planck function, ε is the surface emissivity for the SI-111 radiometer, and $L^{↓}$ is the atmospheric downwelling radiance.

The second part was gathered using an infrared thermometer. As shown in Figure 1, four typical land cover types of subareas were selected in this study: subarea 1 represents vegetation, subarea 2 represents water bodies, subarea 3 represents bare soil, and subarea 4 represents ISA. To obtain hourly LST measurements for each subarea, four Testo 381 infrared thermometers (accuracy: ±1.5 °C) were used to measure LST at a height of 10 cm height. Land cover type changes among vegetation, water bodies, bare soil, and ISA were recorded 60 times per hour on 11 August 2019.

### 2.3. Methods

The premise of spatiotemporal fusion is that LST data retrieved from different satellite sensors on the same date are consistent and comparable [33]. However, discrepancies may arise due to variations in sensor systems, such as differences in satellite overpass times, solar geometry, and bandwidth. The primary objective of the proposed framework in this study is to fuse high spatiotemporal LST data from FY-4A, MOD11A1, and Landsat. Therefore, for the purposes of this study, the LST data from these satellite sensors were assumed to represent simultaneous observations.

**Figure 3:**
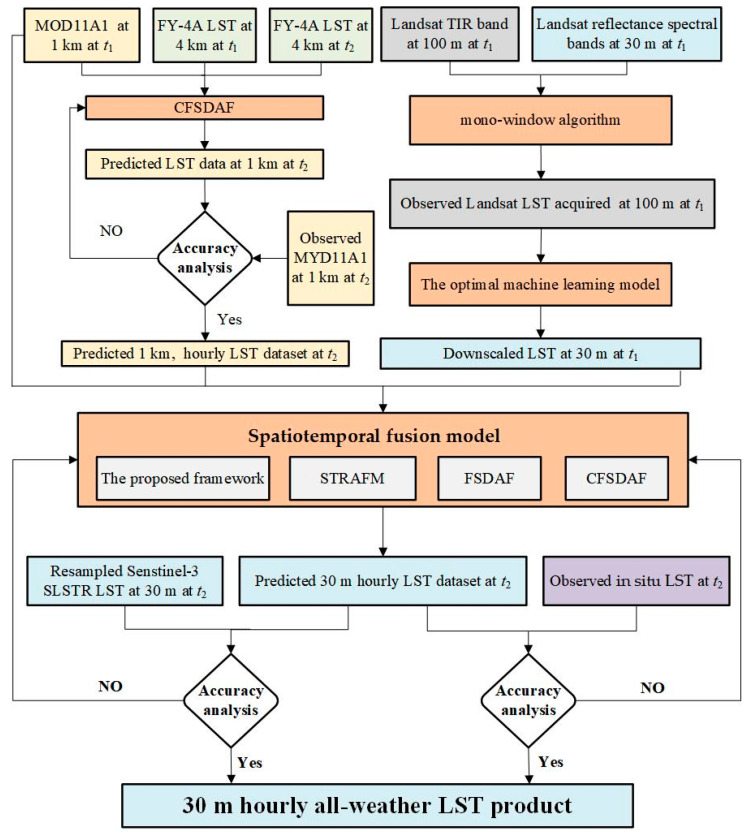
Flowchart of the proposed framework with FY-4A, MOD11A1, and downscaled LST data.

In this study, two steps need to be implemented to predict the 30 m hourly LST data set (Figure 3). In the first step, two pairs of MOD11A1 and FY-4A LST at 11:00 local time on 11 August 2019 and 21 April 2021, respectively, were used as the input base time (*t*_1_) LST data for the spatiotemporal fusion model. At the same time, the other hourly FY-4A LST data (from 00:00 to 10:00 and 12:00 to 23:00 local time) on the same date were used as the input prediction time (*t*_2_) LST data for predicting hourly LST dataset at 1 km spatial resolution. In the following part, LST data directly from FY-4A, MODIS, Landsat LST, Sentinel-3 LST, and in situ LST data were called “observed LST”, and the predicted LST from the proposed framework and other fusion methods were called “predicted LST”. To test the performance of the predicted 1 km hourly LST dataset, the observed MYD11A1 LST data at *t*_2_ (14:00 local time) were used for validation. The coefficient of determination (*R*^2^), root-mean-square error (RMSE), estimated biases, absolute average difference (AAD), and Moran’s I index were calculated to compare the observed MOD11A1 data and the predicted LST. If the first part demonstrates satisfactory performance, we can proceed to the next step.

**Figure 4:**
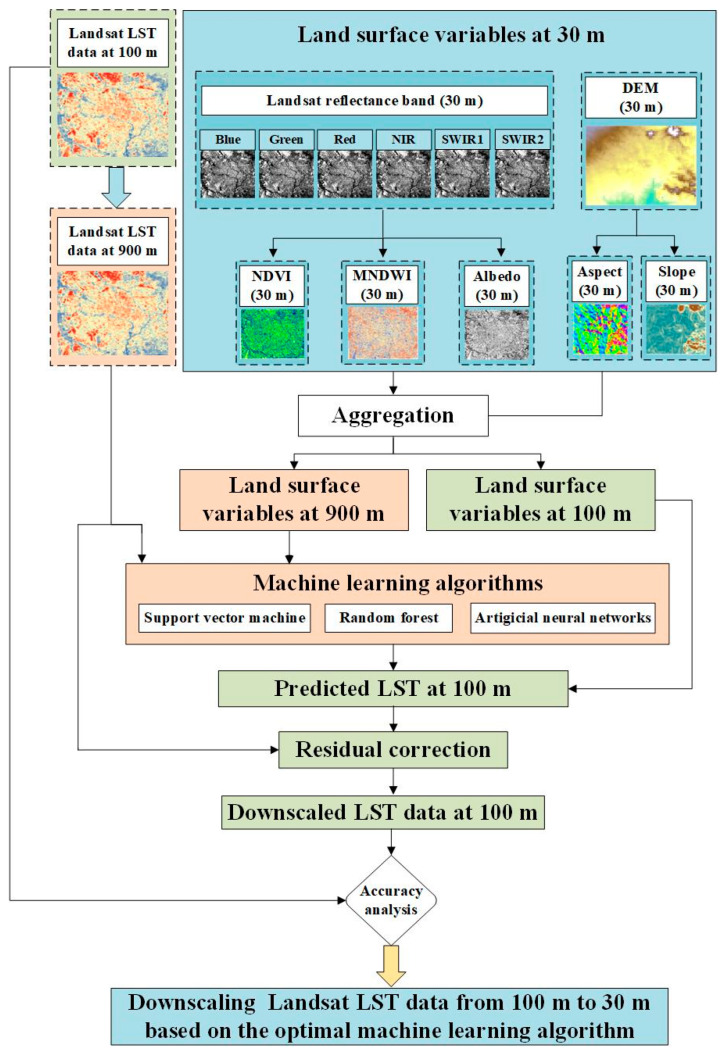
Flowchart of LST downscaling procedure using the machine learning methods.

In the second step, the optimal machine learning model (Figure 4) was selected to downscale Landsat LST at *t*_1_ from 100 m to 30 m. By combining downscaled LST and MOD11A1 data at *t*_1_ with the predicted 1 km hourly LST dataset from the first step at *t*_2_, a fused LST dataset with 30 m spatial resolution and 1 h temporal resolution was generated using a spatiotemporal fusion model.

#### 2.3.1. Comprehensive Flexible Spatiotemporal Data Fusion (CFSDAF)

The Flexible Spatiotemporal DAta Fusion (FSDAF) method is the most popular approach for spatiotemporal fusion of LST due to minimum data requirements and can effectively predict both gradual and abrupt change events simultaneously [63,64,65]. However, FSDAF can easily lead to spatial discontinuities in LST due to the hard classification applied to the high spatial resolution data, making it less feasible for use in heterogeneous urban areas. Shi et al. [66] proposed a comprehensive FSDAF (CFSDAF) method, which can preserve the spatial details and continuity of LST in urban areas.

The CFSDAF model can be expressed as follows:

(2) $${\tilde{LST}}_{t_{2}}(x_{ij},y_{ij})={LST}_{t_{1}}(x_{ij},y_{ij})+\sum_{k=1}^{n}\left[w_{k}\times ∆LST(x_{ij},y_{ij})\right]$$

where ${\tilde{LST}}_{t_{2}}(x_{ij},y_{ij})$ is the predicted LST data with high spatial resolution. ${\;LST}_{t_{1}}(x_{ij},y_{ij})$ represents the input base LST data with high spatial resolution at $t_{1}$. $∆LST\left(x_{ij},y_{ij}\right)$ is the final increment by integrating the predicted increments and the residual for one high spatial resolution pixel. *k* is the *k*th similar pixel. $w_{k}$ is the weight for *k*. *n* represents the number of similar pixels to the central pixel within a sliding window. For more details on the CFSDAF model, refer to Shi et al. [66].

The CFSDAF method in this study was carried out in six main steps: (1) adjust for differences between high and low spatial resolution LST data; (2) extract the members and classify high spatial resolution LST data; (3) get the temporal increments through a spatial unmixing process; (4) get the spatial increments using inverse distance weighting (IDW) interpolation; (5) combine the temporal and spatial increments; and (6) obtain the predicted LST data by the information of neighborhood.

#### 2.3.2. Downscaling LST Using Machine Learning Algorithm

In this study, three widely used machine learning algorithms—SVM [67], random forest (RF) [68], and artificial neural networks (ANNs) [69]—were employed for downscaling Landsat LST data from 100 m to 30 m at time *t*_1_. These algorithms were selected due to their effectiveness in LST downscaling tasks, where they are well-regarded for handling non-linear relationships, complex patterns, and various spatial dependencies within the data.

(1) SVM is a widely used machine learning method rooted in statistical learning theory, especially effective for handling complex nonlinear relationships within data and high-dimensional pattern recognition tasks [70]. In essence, SVM is highly suitable for both classification and regression challenges in remote sensing studies [71]. A range of kernel functions, such as linear, radial basis function, and polynomial have been introduced to enhance SVM performance. In this study, the e1071 package in R software was used for LST downscaling; the optimal values for the penalty parameter (C) and gamma (γ) were determined by performing 10-fold cross-validation to minimize model error.

(2) RF is an advanced machine-learning algorithm that provides high accuracy and robustness by utilizing a large number of random and uncorrelated decision trees [72]. Decision trees are built by generating random subsets of the training data through bootstrap sampling. Variables not included in the subset form the “Out-of-Bag” (OOB) samples, which are used for model performance evaluation. As a nonlinear ensemble regression method, RF is composed of a set of uncorrelated classification and regression trees. These trees are based on the Classification and Regression Trees (CART) algorithm [73]. In this study, two key parameters—ntree (the number of decision trees) and mtry (the number of predictor variables considered at each node)—are optimized to minimize model error and improve the accuracy of the constructed model. The optimal values for ntree and mtry were simultaneously determined using a 10-fold cross-validation approach.

(3) ANN is a class of statistical learning algorithms introduced over half a century ago and has since been successfully applied for downscaling in various fields [74]. ANN is effective at addressing nonlinear problems and modeling complex relationships between variables across different scales. It consists of three layers: input, output, and one or more hidden layers [75]. Neurons are the basic units, and weights represent connections between them. The learning rule adjusts the weights and connections based on input/output data, enabling the model to improve its accuracy. The number of nodes at the hidden layer, moment coefficient, learning rate, and number of iterations were used to train the method procedure.

The above downscaling approach leverages the relationship between LST and associated land surface variables [76]. Assuming that the relationship between LST and three variables is scale-invariant, the process involves constructing this relationship at a low spatial resolution and applying it to the study area at a high spatial resolution. The selected variables include a Digital Elevation Model (DEM) and its derivatives (slope and aspect) with a spatial resolution of 30 m, derived from NASA’s Shuttle Radar Topography Mission (SRTM). Additional variables include the Normalized Difference Vegetation Index (NDVI), Modified Normalized Difference Water Index (MNDWI), albedo, reflectance bands, and land cover information.

The specific steps of the LST downscaling process are illustrated in Figure 3.

(1) Currently, there are no actual LST datasets with 30 m spatial resolution covering large-scale areas. Therefore, to evaluate the performance of the three machine learning methods in downscaling LST data, the observed Landsat LST at 100 m resolution was aggregated to 900 m in order to preserve the multiple relationships with the original Landsat LST data. The land surface variables were aggregated to 900 m as well, the relationship between LST and the land surface variables at 900 m can be expressed as follows:

(3) $${\mathrm{LST}}_{900}=f({NDVI}_{900},{{MNDWI}_{900},{Albedo}_{900},{Refelectance\;band}_{900},DEM}_{900},{Slope}_{900},{Aspect}_{900})$$

where *f*(.) is a nonlinear regression model between LST and land surface variables at 900 m.

(2) The LST residual information at 900 m spatial resolution, ${∆LST}_{l}$, can be expressed as:

(4) $${∆LST}_{l}={LST}_{l}-\bar{{LST}_{l}}$$

where $\bar{{LST}_{l}}$ is the LST data with low spatial resolution generated by the machine learning method and ${LST}_{l}$ represents the LST at 100 m spatial resolution.

(3) The downscaled LST with 100 m resolution can be derived as follows:

(5) $${\mathrm{LST}}_{100}=f({NDVI}_{100},{{MNDWI}_{100},{Albedo}_{100},{Refelectance\;band}_{100},DEM}_{100},\;{Slope}_{100},{Aspect}_{100})+\;{∆LST}_{l}$$

(4) The downscaled LST data with 100 m spatial resolution, produced using three machine learning algorithms, were validated against the observed Landsat LST with 100 m spatial resolution. The *R*^2^ and RMSE were used to assess their performance, and the optimal downscaling method was selected to further downscale observed Landsat LST from 100 m to 30 m, following the same procedure.

## 3. Results

### 3.1. Spatiotemporal Fusion of FY-4A LST and MOD11A1 Data

**Figure 5:**
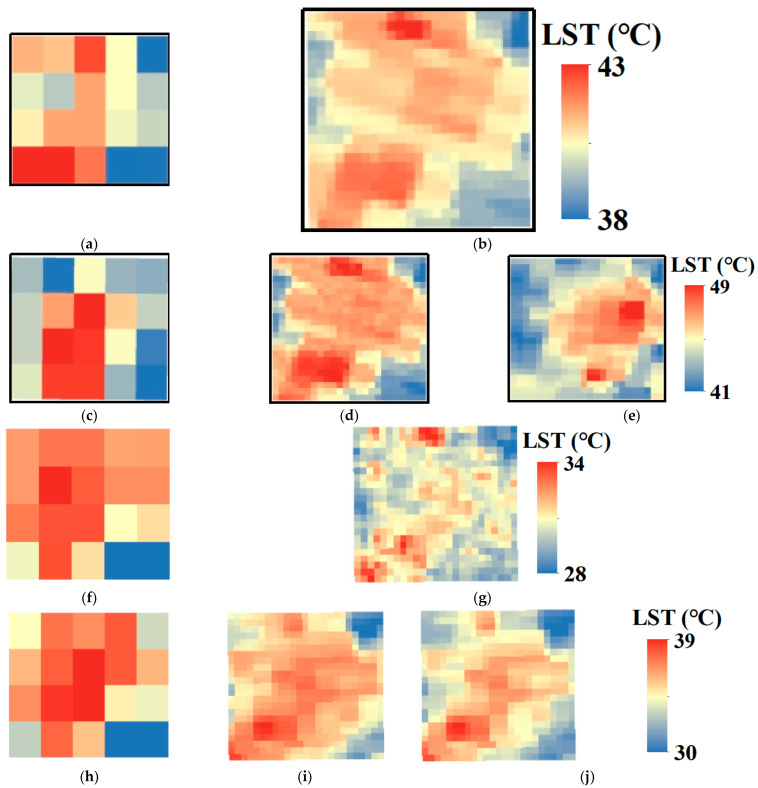
Comparison between the observed LST and predicted LST. (**a**) observed FY-4A LST, 11:00 local time, 11 August 2019. (**b**) observed MOD11A1, 11:00 local time, 11 August 2019. (**c**) observed FY-4A LST, 14:00 local time, 11 August 2019. (**d**) predicted LST, 14:00 local time, 11 August 2019. (**e**) observed MYD11A1, 14:00 local time, 11 August 2019. (**f**) observed FY-4A LST, 11:00 local time, 21 April 2022. (**g**) observed MOD11A1, 11:00 local time, 21 April 2022. (**h**) observed FY-4A LST, 14:00 local time, 21 April 2022. (**i**) predicted LST, 14:00 local time, 21 April 2022. (**j**) observed MYD11A1, 14:00 local time, 21 April 2022.

In this experiment, the observed FY-4A LST (Figure 5a,f) and observed MOD11A1 data (Figure 5b,g) at 11:00 local time on 11 August 2019 and 21 April 2022, respectively, were used as the input base LST data of CFSDAF at *t*_1_ for predicting the 1 km hourly LST data at *t*_2_. Figure 5c,h shows the observed FY-4A LST at *t*_2_ (14:00 local time) on the same dates, which were used to predict the 1 km LST at *t*_2_ (Figure 5d,i). The observed MYD11A1 at 14:00 local time on 11 August 2019 (Figure 5e) and 21 April 2022 (Figure 5j) were used to validate the predicted LST results as the corresponding time at *t*_2_.

**Figure 6:**
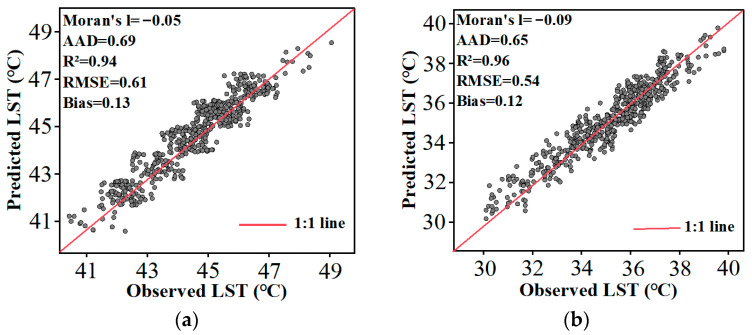
Scatter plot comparison between predicted LST by CFSDAF and observed MYD11A1 LST for (**a**) 14:00 on 11 August 2019 and (**b**) 14:00 on 21 April 2022.

Figure 6 presents the scatter plots illustrating the correlation between observed and predicted LST at 14:00 local time on 11 August 2019 and 21 April 2022. The R² values for both dates exceed 0.9, demonstrating a strong correlation. The RMSE values are 0.61 and 0.54, the AAD values are 0.69 and 0.65, and the biases are 0.13 and 0.12, respectively. The Moran’s I index values are all close to 0, indicating that the predicted LST errors are randomly distributed in space. The CFSDAF method does not systematically overestimate or underestimate values in specific regions. These results demonstrate that the CFSDAF method effectively combines FY-4A and MOD11A1 data to generate hourly LST datasets.

**Figure 7:**
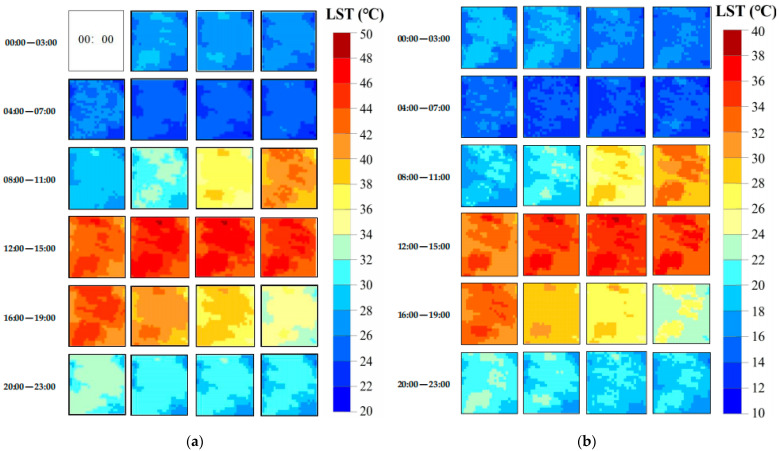
The generated 1 km hourly LST dataset: (**a**) 11 August 2019, and (**b**) 21 April 2022.

Therefore, the observed FY-4A LST data from 00:00 to 23:00 local time (excluding 11:00 local time) were used as the input base LST data at *t*_2_ to predict hourly LST using the CFSADF model for 11 August 2019 and 21 April 2022. This process generated 1 km hourly LST data (including the observed LST at 11:00 local time) for the study area on 11 August 2019 (Figure 7a) and 21 April 2022 (Figure 7b).

### 3.2. Spatiotemporal Fusion of Downscaled LST Data, FY-4A LST, and MOD11A1

**Figure 8:**
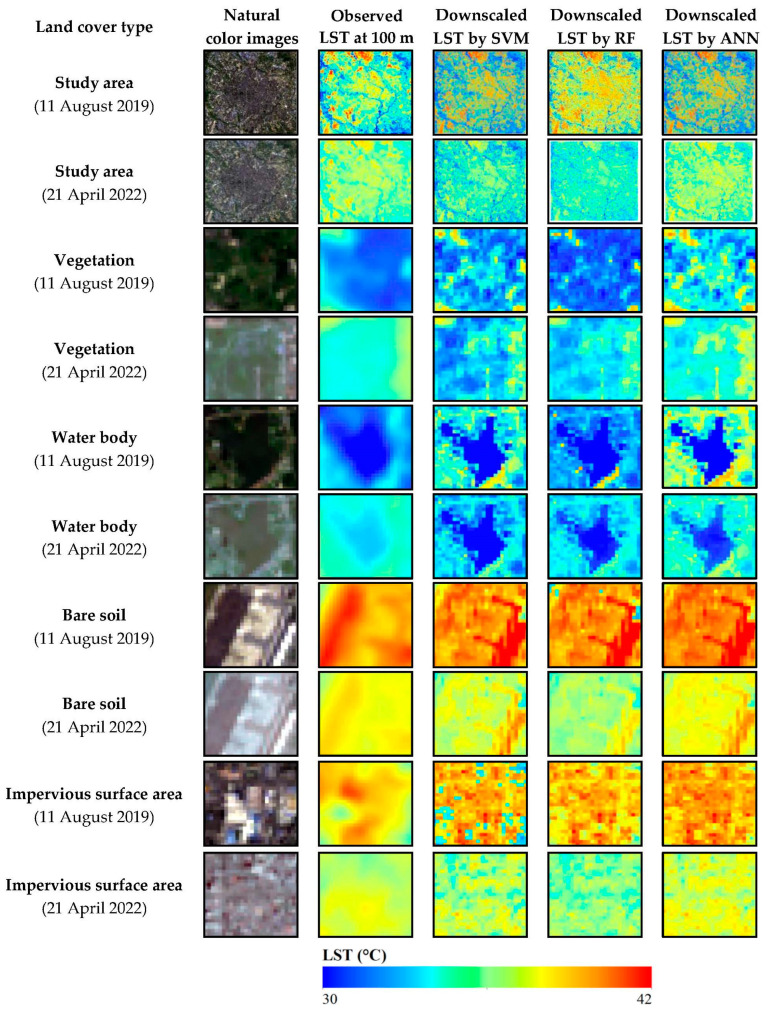
Comparison between observed 100 m Landsat 8 LST and downscaled LST with 100 m spatial resolution using machine learning algorithms.

Machine learning algorithms, including SVM, RF, and ANN, were utilized to downscale the aggregated Landsat 8 LST data at *t*_1_ from 900 m to 100 m to evaluate the performance of each downscaling method (Figure 8). The required parameterizations are detailed as follows:

(1) For the SVM, the Radial Basis Function (RBF) kernel was selected in this study. To minimize errors in the SVM method, the penalty parameter (C) was set to range from 8 to 64 and gamma (γ) from 32 to 128. These parameters were optimized using a grid search method combined with 10-fold cross-validation, where all pixels were divided into ten groups, with eight groups used for training and the remaining two for validation in each iteration; (2) For the RF, the parameters ntree and mtry were automatically determined through 10-fold cross-validation. After optimization, ntree = 500 and mtry = 3 were found to be sufficient for this study; (3) For the ANN, the relatively optimal structural parameters were selected based on multiple tests. These included the number of nodes in the first hidden layer (ranging from 5 to 50), the momentum coefficient (ranging from 0 to 1), the learning rate (ranging from 0 to 1), and the number of iterations (ranging from 100 to 50,000).

Figure 8 presents the spatial distribution of downscaled 100 m LST covering the entire study area, as well as four land cover types. Compared with the spatial distribution of observed LST, the detailed features of the downscaled LSTs are preserved completely and closely resemble the observed LST. The downscaled LST of the RF algorithm outperforms those of SVM and ANN in the study area. In land cover types, SVM exhibits clear underestimations, while ANN shows overestimations for both vegetation and ISA types. The downscaled LST results were validated against the observed LST data. As shown in Table 3, in terms of downscaling accuracy, RF is universally better than other methods in study areas, vegetation types, water bodies, and ISA types. The downscaling accuracy of ANN is highest for bare soil types. Additionally, the downscaling accuracy in regions with fully water bodies and vegetation is superior to that in heterogenous areas, such as ISA and bare soil. This is attributed to the strong association between the spatial distribution of LST and water indices, such as MNDWI, as well as vegetation indices and NDVI. In summary, this study selected RF as the optimal machine learning downscaling algorithm for subsequent research.

**Figure 9:**
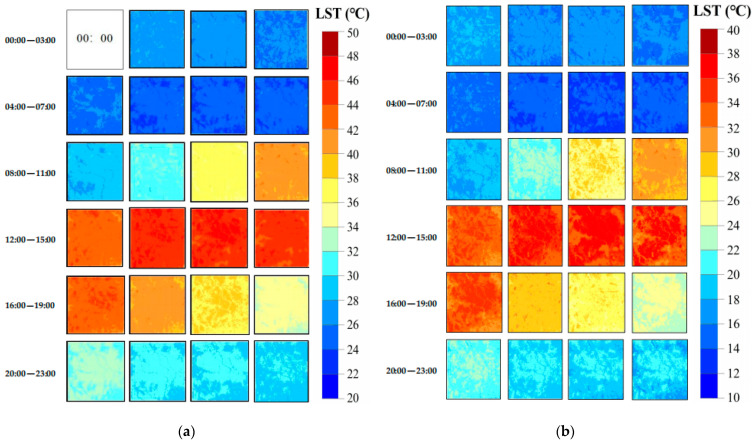
The generated 30 m hourly LST dataset: (**a**) 11 August 2019, and (**b**) 21 April 2022.

Therefore, the downscaled LST with 30 m spatial resolution using RF, along with the 1 km MOD11A1 data at *t*_1_ and 1 km hourly LST data at *t*_2_, were used as the input base LST data for the CFSDAF method to predict the 30 m hourly LST data on 11 August 2019 (Figure 9a), and 21 April 2022 (Figure 9b).

**Figure 10:**
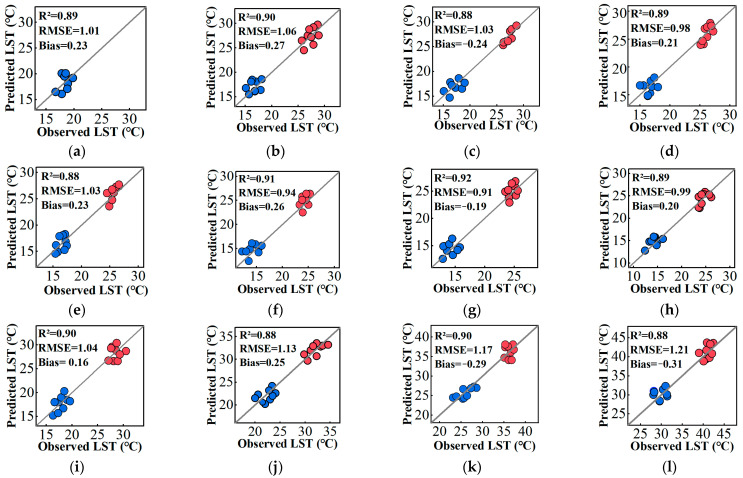

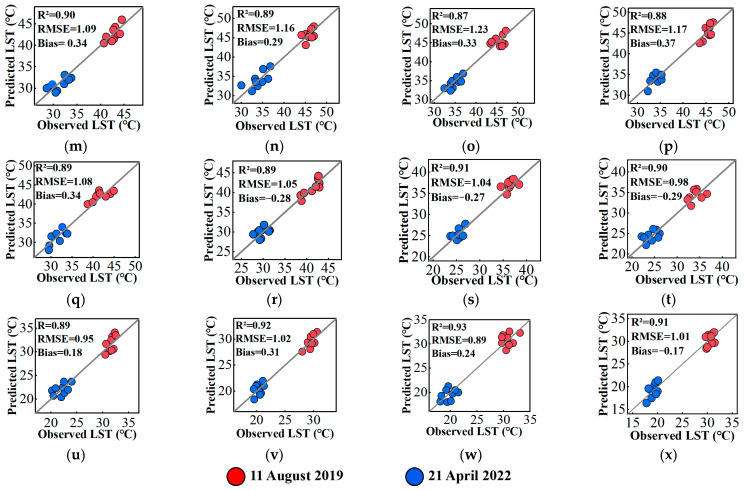
Scatter plots of the relationship between the predicted LST results and observed in situ LST on 11 August 2019 and 21 April 2022: (**a**) 00:00; (**b**) 01:00; (**c**) 02:00; (**d**) 03:00; (**e**) 04:00; (**f**) 05:00; (**g**) 06:00; (**h**) 07:00; (**i**) 08:00; (**j**) 09:00; (**k**) 10:00; (**l**) 11:00; (**m**) 12:00; (**n**) 13:00; (**o**) 14:00; (**p**) 15:00; (**q**) 16:00; (**r**) 17:00; (**s**) 18:00; (**t**) 19:00; (**u**) 20:00; (**v**) 21:00; (**w**) 22:00; (**x**) 23:00.

Since there are no satellite TIR sensors that can derive hourly LST data at 30 m spatial resolution, in situ hourly LST measurements from nine weather stations were used to validate the predicted results using the proposed framework (Figure 10). As shown in Figure 10, the *R*^2^ between the predicted LST and observed in situ LST ranges from 0.87 to 0.93, with RMSE and bias values ranging from 0.70 °C to 1.47 °C and 0.16 °C to 0.37 °C, respectively. These accuracy assessment results demonstrate that the proposed framework achieves higher accuracy in generating high spatial resolution hourly LST data.

Additionally, the traditional spatiotemporal fusion models, such as STARFM, FSDAF, and CFSDAF were also used to validate the performance of the proposed framework. As seen in Table 4, under the same spatiotemporal fusion workflow, The *R*^2^, RMSE, and bias between the predicted 30 m hourly LST from the proposed framework and 100 m hourly LST from the traditional spatiotemporal fusion model, respectively, with observed in situ LST also demonstrate that the proposed framework generates more accurate hourly LST data compared to other methods.

**Figure 11:**
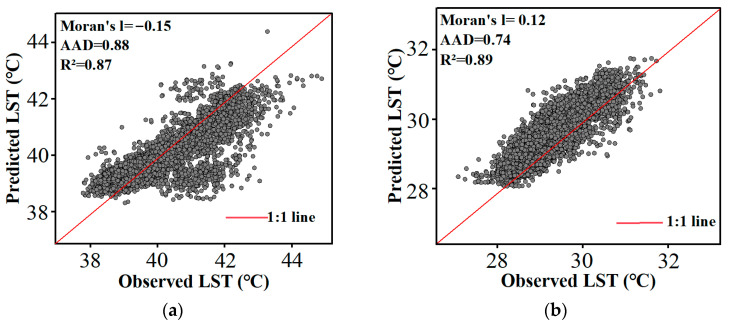

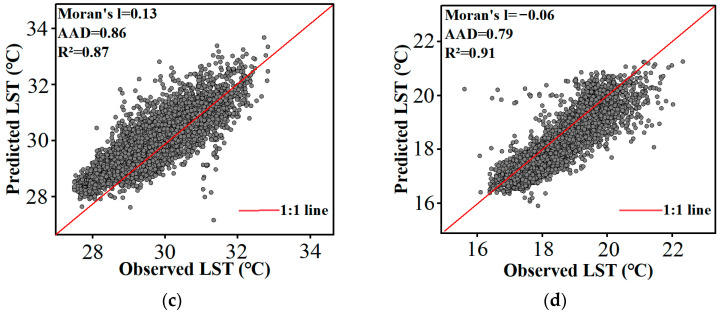
Scatter plots of predicted LST results against observed Sentinel-3 SLSTR LST product: (**a**) 11:00 local time on 11 August 2019, (**b**) 23:00 local time on 11 August 2019, (**c**) 11:00 local time on 21 April 2022, and (**d**) 23:00 local time on 21 April 2022.

**Figure 12:**
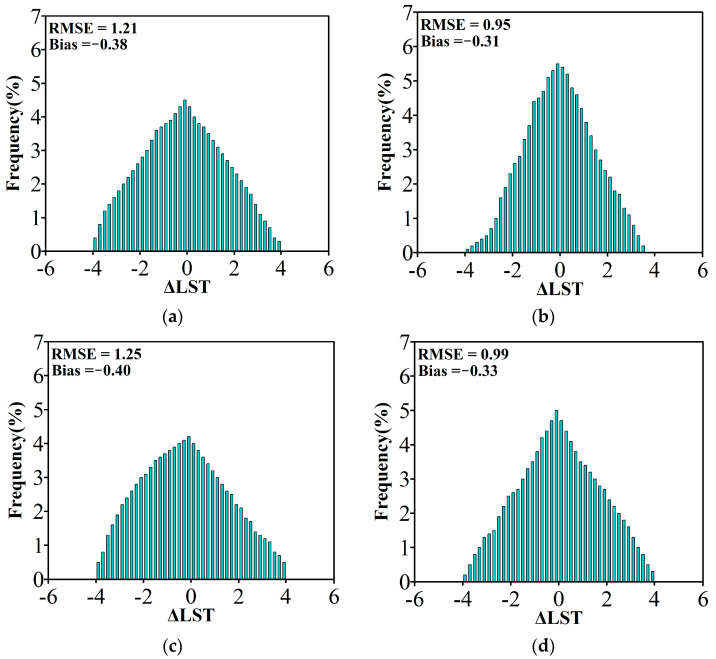
Distribution of LST errors between the predicted LST results and observed Sentinel-3 SLSTR LST product: (**a**) 11:00 local time on 11 August 2019, (**b**) 23:00 local time on 11 August 2019, (**c**) 11:00 local time on 21 April 2022, and (**d**) 23:00 local time on 21 April 2022.

Considering the limited number of meteorological stations, two pairs of Sentinel-3 SLSTR LST products with 1 km spatial resolution (acquired at 11:00 local time and 23:00 local time) on 11 August 2019 and 21 April 2022 were used for cross-validation to further validate the accuracy of the LST predicted by the proposed framework. Sentinel-3 SLSTR LST product has been released by the European Satellite Agency (https://dataspace.copernicus.eu/ accessed on 5 August 2024), Recently, many scholars have discovered that the RMSE between SLSTR LST and in situ LST was about 2–3 K, and the bias was approximately 1–2 K [77,78,79,80]. To ensure consistency in spatial resolution, the Sentinel-3 SLSTR LST products were resampled to 30 m. Figure 11 shows a good agreement between the predicted LST and the Sentinel-3 SLSTR LST product, with the *R*^2^ ranging from 0.87 °C to 0.89 °C, AAD ranging from 0.74 °C to 0.88 °C, and the Moran’s I index values are all close to 0. The residual histograms comparing the predicted LST with the observed Sentinel-3 SLSTR LST product are presented in Figure 12. The results indicate that the RMSE ranges from 0.95 to 1.25, while the biases are nearly zero, demonstrating the unbiased predicted LST achieved by the proposed framework.

## 4. Discussion

### 4.1. Predicted Hourly LST Data for Different Land Cover Types

**Figure 13:**
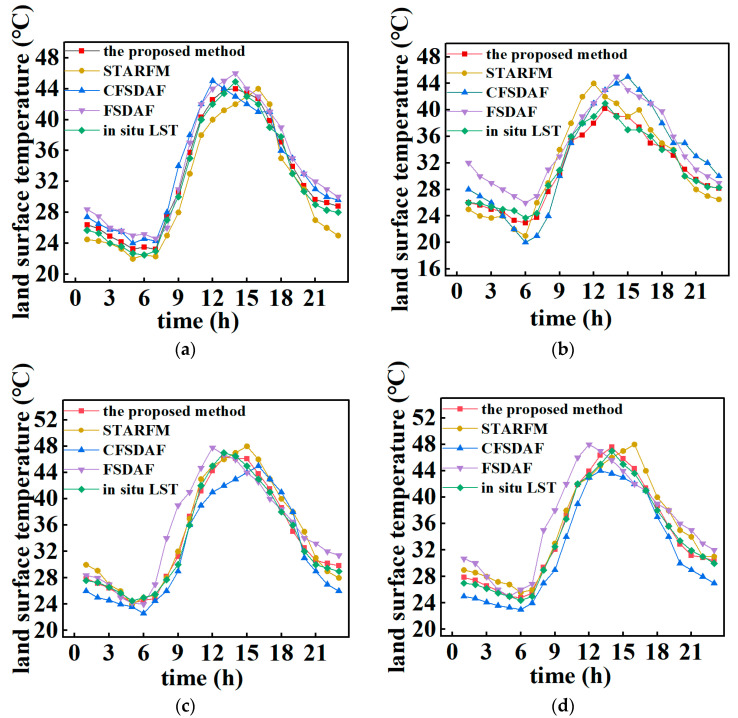
Comparison of the predicted hourly LSTs using the proposed framework and the predicted 100 m hourly LSTs using STARFM, FSDAF, and CFSDAF, respectively, with observed in situ LST using infrared thermometer on 11 August 2019. (**a**) subarea 1 (vegetation). (**b**) subarea 2 (water body). (**c**) subarea 3 (bare soil). (**d**) subarea 4 (ISA).

**Figure 14:**
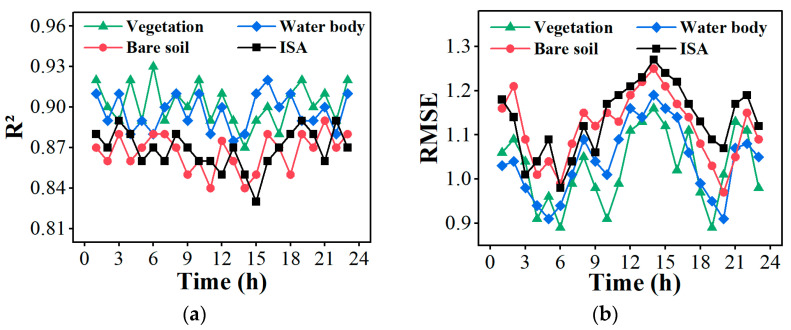
(**a**) *R*^2^ and (**b**) RMSE between the predicted hourly LSTs using the proposed framework and the observed in situ LST using infrared thermometer on 11 August 2019.

Figure 13 further illustrates the variations in in situ hourly LST for each land cover type obtained from field measurements using an infrared thermometer alongside the predicted hourly LST data generated by the proposed framework and other spatiotemporal fusion models. It is evident that the predicted hourly LST time variation curve generated by the proposed framework in this study fits the in situ hourly LST best, while other methods exhibit significant discrepancies across different land cover types during specific time periods. It was shown that *R*^2^ between the predicted hourly LST and in situ hourly LST were >0.84 for different land cover types (Figure 14), except for ISA type. RMSE was <1.27 for all land cover types. Overall, the accuracy is higher for vegetation and water bodies, while the accuracy for bare soil and ISA is relatively lower. This may be attributed to the presence of mixed pixels in complex urban areas, which can affect the predicted LST for bare soil and ISA types [81]. LST estimation methods often overlook the influence of land use and land cover (LULC) composition within each pixel. In fact, pixels containing multiple types of LULC are classified as mixed pixels, a phenomenon commonly observed in urban thermal environment monitoring [82].

### 4.2. The Applicability of the Proposed Framework in Other Regions

**Figure 15:**
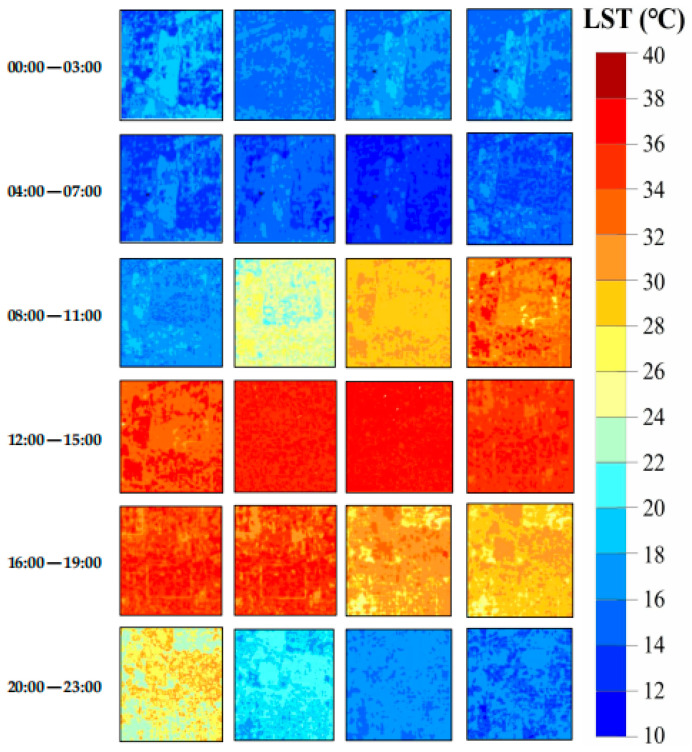
The generated 30 m hourly LST dataset using the proposed framework in Xi’an on 7 April 2022.

To further assess the applicability of the proposed framework in other cities, this study selected Xi’an, the city with the highest urbanization rate in northwest China, as the study area. Using the framework presented in this study, a 30 m hourly LST dataset (Figure 15) was predicted and generated employing the same data sources and processing workflow. From Table 5, the *R*^2^ values between the predicted LST and the Sentinel-3 SLSTR LST product at 11:00 and 23:00 local time are 0.90 and 0.89, respectively. The RMSE ranges from 1.25 to 1.33, while the AAD ranges from 0.74 to 0.85 during the same period. These results indicate that the proposed framework demonstrates strong performance in predicting hourly LST data in other urban areas.

### 4.3. Limitations and Prospects of the Proposed Framework

The proposed framework in this study can be applied in the following areas: agricultural and forestry monitoring [83], surface urban heat island monitoring [84], public health management [85], and high spatiotemporal resolution of land surface temperature and sea surface temperature generated [86]. Although the proposed framework can predict high spatial and temporal LST data, it also has certain limitations. First, the proposed framework in this study is designed for clear-sky conditions, the input LST data for the proposed method are cloud-free data. The existing reconstruction algorithms for LST under cloudy conditions can be grouped into five categories: spatiotemporal gap-filling methods [87], temporal gap-filling methods [88], spatial gap-filling methods [89], surface energy balance-based gap-filling methods [90], and multi-source fusion-based gap-filling methods [91]. In future studies, it is important to integrate reconstruction algorithms with spatiotemporal fusion methods, which offer a promising approach for producing all-weather data coverage across most regions globally. Second, the urban thermal environment is frequently complex in nature, and the complexity depends on numerous factors, such as the heterogeneous structure of the land surface. It is essential to integrate the land surface temperature spatiotemporal fusion method with an approach for pixel decomposition to enhance the accuracy of the predicted high spatiotemporal resolution LST data in urban areas. Third, integrating this study with deep learning to predict LST products while addressing training challenges caused by limited spectral richness is essential. Optimizing sample selection and designing comprehensive dictionary pairs to capture spectral, spatial, temporal, and structural features while maximizing detail preservation is critical for advancing spatiotemporal fusion.

## 5. Conclusions

In this study, a framework combining the CFSDAF model and RF algorithm was proposed and successfully applied to obtain hourly LST with 30 m spatial resolution from FY-4A, MOD11A1, and Landsat LST data. The predicted LST demonstrated relatively strong accuracies, with *R*^2^ and RMSE values ranging from 0.87 °C to 0.93 °C and 0.89 °C to 1.23 °C, respectively, as evaluated against the observed in situ LST data. Compared to traditional spatiotemporal fusion methods, the proposed framework achieves higher accuracy in generating high spatial resolution hourly LST data and exhibits good applicability across different land cover types. The predicted LST data with high spatial resolution and frequent coverage will be beneficial for monitoring diurnal environmental dynamics at various spatial scales.

## Figures and Tables

**Table 1:** Summary of accuracy (%), kappa statistics, area statistics of SVM maps for 2019 and 2022.

Land Cover Type	2019				2022			
	User’s Accuracy	Producer’s Accuracy	Area (km^2^)	Percentage (%)	User’s Accuracy	Producer’s Accuracy	Area (km^2^)	Percentage (%)
Vegetation	0.92	0.94	253.00	49.30	0.93	0.95	203.00	39.60
Water body	0.90	0.93	5.00	1.00	0.92	0.94	4.00	0.80
Bare soil	0.89	0.92	19.00	3.70	0.90	0.93	10.00	1.90
ISA	0.87	0.90	236.00	46.00	0.88	0.91	296.00	57.70
Overall accuracy	0.92				0.94			
Kappa statistics	0.87				0.90			
Total	/	/	513.00	100.00	/	/	513.00	100.00

**Table 2:** The details of the land surface temperature data used in this study.

Data	Spatial Resolution	Temporal Resolution	Acquisition Data	Acquisition Time (Local Time)
FY-4A	4 km	1 h	11 August 2019/ 21 April 2022	Starting at 00:00 and every hour thereafter
MOD11A1	1 km	1 day	11 August 2019/ 21 April 2022	11:00
Landsat 8	100 m	16 days	11 August 2019	11:30
Landsat 9	100 m	16 days	21 April 2022	11:30
In situ measured LST	/	1 h	11 August 2019/ 21 April 2022	Starting at 00:00 and every hour thereafter

**Table 3:** Downscaling performance statistics for SVM, RF, and ANN over the study area on 11 August 2019 and 21 April 2022.

Area	SVM (11 August 2019/ 21 April 2022)		RF (11 August 2019/ 21 April 2022)		ANN (11 August 2019/ 21 April 2022)	
	*R* ^2^	RMSE	*R* ^2^	RMSE	*R* ^2^	RMSE
Study area	0.88/0.90	1.10/1.14	0.90/0.89	1.05/1.12	0.86/0.91	1.20/1.12
Vegetation	0.92/0.89	0.75/1.02	0.93/0.92	0.65/1.03	0.91/0.89	0.80/0.97
Water body	0.89/0.88	0.85/0.96	0.90/0.93	0.78/0.98	0.87/0.87	0.90/0.91
Bare soil	0.84/0.88	1.25/1.21	0.86/0.89	1.15/1.21	0.87/0.90	1.10/1.21
ISA	0.85/0.89	1.20/1.14	0.88/0.86	0.91/0.97	0.83/0.88	1.30/1.24

**Table 4:** Comparison of the predicted 30 m hourly LSTs using the proposed framework and the predicted 100 m hourly LSTs using STARFM, FSDAF, and CFSDAF, respectively, with observed in situ LST on 11 August 2019 and 21 April 2022.

Time (Local Time)	*R* ^2^				RMSE				Bias			
	A	B	C	D	A	B	C	D	A	B	C	D
00:00	0.89	0.88	0.87	0.88	1.01	1.16	1.21	1.17	0.23	0.33	−0.29	0.31
01:00	0.90	0.87	0.86	0.87	1.06	1.11	1.16	1.21	0.27	−0.37	0.34	0.33
02:00	0.88	0.86	0.85	0.86	1.03	1.18	1.22	1.23	−0.24	0.33	0.36	0.34
03:00	0.89	0.87	0.88	0.88	0.98	1.16	1.15	1.19	0.21	0.36	−0.31	−0.29
04:00	0.88	0.85	0.87	0.86	1.03	1.21	1.08	1.16	0.23	0.32	−0.27	−0.28
05:00	0.91	0.87	0.88	0.86	0.94	1.19	1.12	1.17	0.26	−0.28	0.29	−0.31
06:00	0.92	0.86	0.85	0.87	0.91	1.21	1.09	1.13	−0.19	0.33	0.31	0.26
07:00	0.89	0.88	0.86	0.86	0.99	1.25	1.26	1.07	0.20	−0.35	−0.33	0.28
08:00	0.90	0.87	0.87	0.88	1.04	1.17	1.22	1.18	0.16	−0.37	−0.34	−0.31
09:00	0.88	0.88	0.84	0.85	1.13	1.26	1.28	1.22	0.25	0.29	−0.29	−0.32
10:00	0.90	0.86	0.85	0.86	1.17	1.28	1.31	1.24	−0.29	0.36	0.35	−0.31
11:00	0.88	0.85	0.83	0.84	1.21	1.31	1.34	1.17	−0.31	−0.38	0.37	0.36
12:00	0.90	0.83	0.82	0.85	1.09	1.34	1.38	1.25	0.34	−0.33	−0.34	0.41
13:00	0.89	0.81	0.82	0.83	1.16	1.32	1.28	1.32	0.29	−0.41	−0.41	−0.39
14:00	0.87	0.84	0.85	0.82	1.23	1.36	1.33	1.35	0.33	0.43	0.43	−0.42
15:00	0.88	0.85	0.84	0.84	1.17	1.38	1.36	1.26	0.37	0.39	0.46	0.36
16:00	0.89	0.87	0.83	0.86	1.08	1.35	1.29	1.28	0.34	0.40	−0.42	0.38
17:00	0.89	0.84	0.85	0.85	1.05	1.34	1.33	1.16	−0.28	−0.38	0.39	0.33
18:00	0.91	0.87	0.87	0.87	1.04	1.31	1.26	1.21	−0.27	−0.36	0.37	−0.34
19:00	0.90	0.88	0.88	0.88	0.98	1.28	1.29	1.23	−0.29	0.39	0.41	−0.26
20:00	0.89	0.86	0.87	0.89	0.95	1.26	1.22	1.28	0.18	0.27	−0.36	−0.33
21:00	0.92	0.89	0.89	0.88	1.02	1.21	1.19	1.27	0.31	0.35	0.33	0.31
22:00	0.93	0.88	0.90	0.87	0.89	1.16	1.17	1.19	0.24	−0.29	−0.35	−0.27
23:00	0.91	0.89	0.89	0.89	1.01	1.14	1.21	1.12	−0.17	−0.28	−0.31	−0.29

A: The proposed framework; B: STARFM; C: FSDAF; D: CFSDAF.

**Table 5:** Comparison of predicted LST results against observed Sentinel-3 SLSTR LST product in Xi’an City at 11:00 and 23:00 local time on 7 April 2022.

Time (Local Time)	*R* ^2^	RMSE	AAD	Bias
11:00 on 7 April 2022	0.90	1.25	0.85	0.38
23:00 on 7 April 2022	0.89	1.33	0.74	−0.31

## Data Availability

Data are contained within the article.
